# Pattern of inflammatory immune response determines the clinical course and outcome of COVID-19: unbiased clustering analysis

**DOI:** 10.1038/s41598-021-87668-z

**Published:** 2021-04-13

**Authors:** Eunyoung Emily Lee, Kyoung-Ho Song, Woochang Hwang, Sin Young Ham, Hyeonju Jeong, Jeong-Han Kim, Hong Sang Oh, Yu Min Kang, Eun Bong Lee, Nam Joong Kim, Bum Sik Chin, Jin Kyun Park

**Affiliations:** 1 Division of Rheumatology, Department of Internal Medicine, Uijeongbu Eulji Medical Center, Gyeonggi-do, Korea; 2 Division of Infectious Diseases, Department of Internal Medicine, Seoul National University Bundang Hospital, Gyeonggi-do, Korea; 3 Data Science for Knowledge Creation Research Center, Seoul National University, Seoul, Korea; 4 Division of Infectious Diseases, Department of Internal Medicine, Armed Forces Capital Hospital, Gyeonggi-do, Korea; 5 Department of Infectious Diseases, Myongji Hospital, Gyeonggi-do, Korea; 6 Department of Medical Education, Seoul National University College of Medicine, Seoul, Korea; 7 Division of Rheumatology, Department of Internal Medicine, Seoul National University College of Medicine, 101 Daehak-ro, Jongno-gu, Seoul, 03080 South Korea; 8 Division of Infectious Diseases, Department of Internal Medicine, Seoul National University Hospital, Seoul, Korea; 9 Division of Infectious Diseases, Department of Internal Medicine, National Medical Center, Euljiro 245, Jung-gu, Seoul, 04564 Korea

**Keywords:** Inflammation, Respiratory tract diseases

## Abstract

The objective of the study was to identify distinct patterns in inflammatory immune responses of COVID-19 patients and to investigate their association with clinical course and outcome. Data from hospitalized COVID-19 patients were retrieved from electronic medical record. Supervised k-means clustering of serial C-reactive protein levels (CRP), absolute neutrophil counts (ANC), and absolute lymphocyte counts (ALC) was used to assign immune responses to one of three groups. Then, relationships between patterns of inflammatory responses and clinical course and outcome of COVID-19 were assessed in a discovery and validation cohort. Unbiased clustering analysis grouped 105 patients of a discovery cohort into three distinct clusters. Cluster 1 (hyper-inflammatory immune response) was characterized by high CRP levels, high ANC, and low ALC, whereas Cluster 3 (hypo-inflammatory immune response) was associated with low CRP levels and normal ANC and ALC. Cluster 2 showed an intermediate pattern. All patients in Cluster 1 required oxygen support whilst 61% patients in Cluster 2 and no patient in Cluster 3 required supplementary oxygen. Two (13.3%) patients in Cluster 1 died, whereas no patient in Clusters 2 and 3 died. The results were confirmed in an independent validation cohort of 116 patients. We identified three different patterns of inflammatory immune response to COVID-19. Hyper-inflammatory immune responses with elevated CRP, neutrophilia, and lymphopenia are associated with a severe disease and a worse outcome. Therefore, targeting the hyper-inflammatory response might improve the clinical outcome of COVID-19.

## Introduction

It is striking that the SARS-CoV-2 virus causes different clinical manifestations, ranging from asymptomatic to mild upper respiratory infection to death due to acute respiratory distress syndrome, in different people^1,2^. Inflammatory markers such as high C-reactive protein (CRP) levels, lymphopenia, and elevated neutrophil counts are associated with a worse outcome^3,4^. Because the immune response is a dynamic process, inflammatory markers change over time, increasing rapidly during the initial phase of infection and falling during the recovery phase. Therefore, it is not a single value at a single time point, but rather longitudinal changes in inflammatory markers, that might better capture individual immune responses that ultimately determine the clinical outcome (i.e., life or death) of patients with COVID-19^5^.

Previously, we reported that common inflammatory markers are not always elevated in patients with systemic rheumatic diseases. Rather, individual autoimmune diseases have a unique profile of inflammatory markers that might be mediated by underlying immune responses that generate a complex and relatively unique set of key cytokines^6,7^. Similarly, different immune responses to COVID-19 might be reflected by a unique profile of inflammatory markers. Also, there is a report suggesting that severe respiratory failure in COVID-19 is driven by a unique pattern of immune dysfunction^8^. Accordingly, we hypothesized that patterns (or subtypes) of inflammatory immune responses are associated with clinical course and outcome. We performed supervised clustering analysis to group COVID-19 patients into distinct clusters based on longitudinal measurement of CRP, absolute neutrophil counts (ANC), and absolute lymphocyte counts (ALC), independent of clinical information such as patient demographics and clinical manifestations. The resulting patterns (clusters) were then examined to identify associations with clinical course and outcome.

Unbiased clustering analysis of routine inflammatory parameters identified a specific immune response pattern that is associated with severe COVID-19 disease and poor outcome.

## Results

### Demographic and clinical characteristics

**Figure 1:**
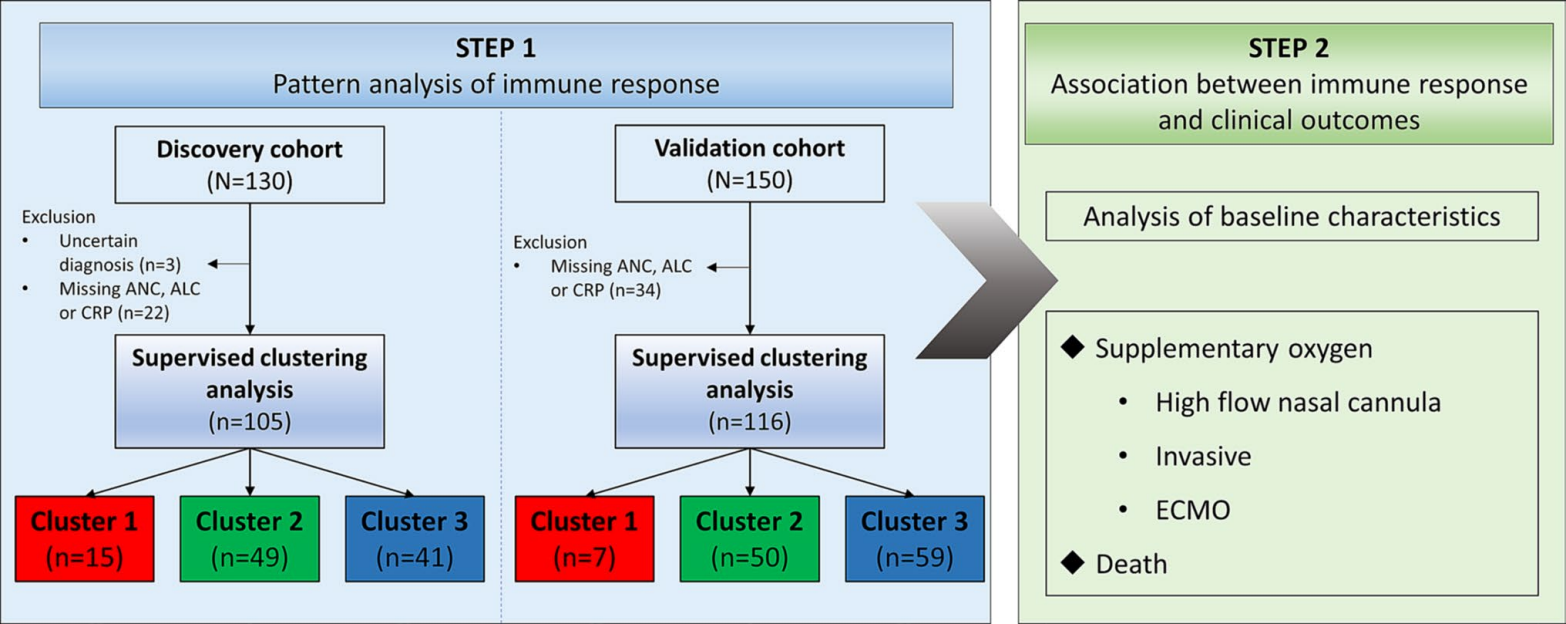
Study design and patient flow. *ALC* absolute lymphocyte count, *ANC* absolute neutrophil count, *CRP* C-reactive protein, *ECMO* extracorporeal membrane oxygenation, *MV* mechanical ventilator.

By 14 April 2020, 130 consecutive patients were recruited from the three medical centers; 78 from NMC, 27 from SNUBH, and 25 from SNUH. Three cases with an uncertain diagnosis and 22 cases with missing data were excluded. Thus, 105 patients were included in the analysis (Fig. 1). The mean age of the patients was 55.2 ± 19.7 years and 62 (59.0%) were male. The time between the first symptom onset and hospitalization was 5.9 ± 4.7 days.

### Clustering of immune responses

**Figure 2:**
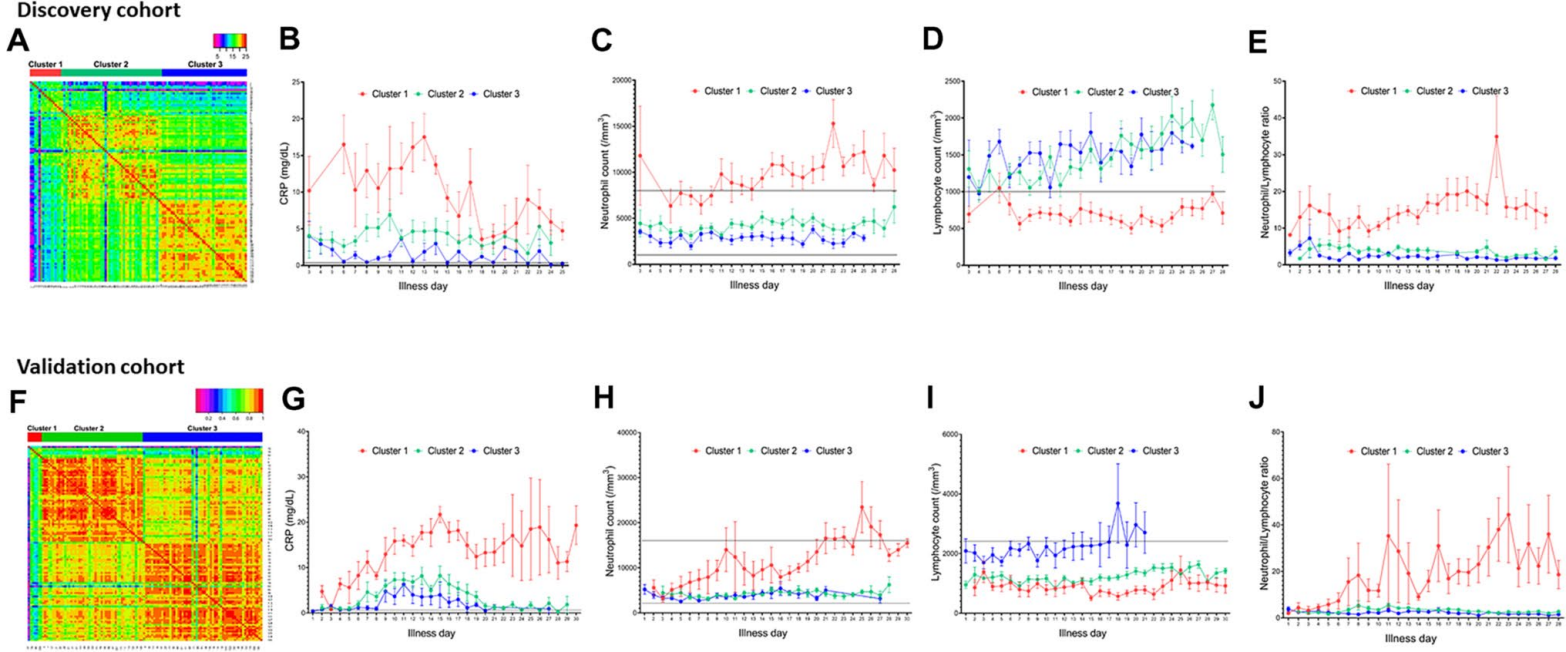
Unbiased clustering of inflammatory responses in those with COVID-19 infection in discovery and validation cohort. Heat map showed three distinct patterns of inflammatory immune response in 105 COVID-19 patients of discovery cohort (**A**) and 116 patients in validation cohort (**F**). Colors indicate the similarity between patients (red = high similarity, violet = low similarity). Changes in C-reactive protein levels in discovery cohort (**B**) and validation cohort (**G**), changes in absolute neutrophil counts in discovery cohort (**C**) and validation cohort (**H**), changes in absolute lymphocyte count in discovery cohort (**D**) and validation cohort (**I**), changes in neutrophil/lymphocyte ratio in discovery cohort (**E**) and validation cohort (**J**) in each cluster are depicted. “Illness day” denotes the number of days from first symptom onset. Horizontal lines indicate the normal ranges. CRP, C-reactive protein. Data are expressed as the mean ± standard error.

Unbiased clustering analysis grouped the 105 patients into three distinct clusters based on temporal measurement of CRP levels, ANC, and ALC (Fig. 2A). The distance indicates the difference or similarity between patterns of inflammatory immune responses in individual patients. CRP levels were highest in Cluster 1, followed by Clusters 2 and 3. In Cluster 1, the mean CRP level peaked at 16.5 mg/dL at the end of the first week of illness and remained high until the end of the second week; this was followed by slow improvement over the following weeks. In Cluster 3, CRP levels remained low over time. Cluster 2 showed a mild elevation of CRP (i.e., < 5 mg/dL) during the early stages (the first 2 weeks of illness) and improved thereafter (Fig. 2B). In Cluster 1, the ANC started to increase and exceeded the upper normal limit by Day 11; it remained high. The ANC in Clusters 2 and 3 remained in normal range (Fig. 2C). Lastly, the ALC in Cluster 1 remained below the reference value. The ALC for Cluster 2 and 3 was close to the lower normal limit during the early stages, but it increased (recovered) slowly over time (Fig. 2D). Taken together, the data show that Cluster 1 had the highest CRP levels, the highest ANC, and the lowest ALC, whereas Cluster 3 had near-normal CRP levels and normal ANC and ALC. Cluster 2 showed an intermediate pattern.

### Baseline demographic and clinical characteristics according to immune response

The baseline demographics and clinical characteristics of the patients included in the analysis are summarized according to cluster (Table 1). Patients in Cluster 1 were significantly older than those in other clusters: mean age, 74.3 ± 9.2, 59.7 ± 15.4, and 43.1 ± 19.8 years in Clusters 1, 2, and 3, respectively. Also, hypertension was more common in Cluster 1 than in Clusters 3 (46.7% vs. 12.2%, p = 0.013 in post hoc multiple comparison). The mean time from first symptom onset to hospitalization was similar across all groups. On admission, white blood cell counts (WBC) in all clusters were normal. However, patients in Cluster 1 had significantly higher ANC, lower ALC and higher CRP levels than those in Clusters 2 and 3. Patients in Cluster 2 and Cluster 3 did not differ statistically in regard to ANC, ALC and CRP at admission (Table 1).

**Table 1:** Baseline characteristics of the patients in the discovery cohort and the validation cohort on admission (according to the clusters).

	Discovery cohort					Validation cohort				
	Total	Cluster 1 (n = 15)	Cluster 2 (n = 49)	Cluster 3 (n = 41)	*p* value*	Total	Cluster 1 (n = 7)	Cluster 2 (n = 50)	Cluster 3 (n = 59)	*p* value*
Male, n (%)	62 (59.0)	10 (66.7)	30 (61.2)	22 (53.7)	0.622	74 (63.8)	3 (42.9)	31 (62.0)	40 (67.8)	0.405
Age, years	55 ± 19.8	74.3 ± 9.2	59.4 ± 15.4	43.1 ± 19.8	< 0.001	50.2 ± 18.6	72.6 ± 10.7	54.9 ± 16.4	43.6 ± 17.9	< 0.001
**Comorbidities, n (%)**										
Hypertension	22 (21.0)	7 (46.7)	10 (20.4)^†^	5 (12.2)	0.019	29 (25.0)	4 (57.1)	18 (36.0)	7 (11.9)	0.001
Diabetes	17 (16.2)	3 (20.0)	11 (22.4)	3 (7.3)	0.138	24 (20.7)	5 (71.4)	14 (28.0)	5 (8.5)	< 0.001
CKD	2 (14.3)	0 (0.0)	2 (4.1)	0 (0.0)	0.312	6 (5.2)	4 (57.1)	2 (4.0)	0	< 0.001
SF ratio	457.1 [342.9;461.9]	232.5 [148.3;308.1]	447.6 [332.1;461.9]	461.9 [457.1;466.7]	< 0.001	461.9 [452.4;466.7]	296.9 [259.7;454.8]	461.9 [442.9;461.9]	461.9 [452.4;466.7]	< 0.001
Mechanical ventilation	4 (3.8)	4 (26.7)	0 (0.0)	0 (0.0)	< 0.001	0 (0.0)	0 (0.0)	0 (0.0)	0 (0.0)	1.000
Time from symptom onset to admission, days	5.9 ± 4.7	6.5 ± 4.2	5.9 ± 4.0	5.8 ± 5.7	0.647	7.1 ± 8.2	4.3 ± 5.8	7.7 ± 7.4	6.9 ± 9.1	0.867
**Inflammatory parameters (reference range)**										
WBC (4.0–10.0), (10^3^/µL)	5.8 ± 2.3	6.9 ± 3.5	5.7 ± 2.0	5.4 ± 2.1	0.052	6.0 ± 2.6	5.8 ± 2.2	5.1 ± 2.3	6.8 ± 2.6	0.004
Neutrophils (50–75), (%)	67.6 ± 14.9	80.4 ± 11.3	68.5 ± 14.4	61.7 ± 13.7^‡^	< 0.001	63.9 ± 14.0	70.2 ± 10.9	64.3 ± 15.2	62.9 ± 13.3	0.242
Lymphocytes (20–40), (%)	22.5 ± 12.0	13.2 ± 9.5	22.0 ± 11.3	26.7 ± 11.7^‡^	< 0.001	25.3 ± 11.3	19.1 ± 9.5	23.8 ± 10.7	27.2 ± 11.7	0.103
ANC (1500–8000), (/µL)	4078 ± 2264	5788.7 ± 3317	4061.4 ± 1929	3457.7 ± 1867^‡^	0.001	4009.6 ± 2317	4183.6 ± 1840	3455.3 ± 2241	4458.7 ± 2362	0.095
ALC (1000–4800), (/µL)	1153.3 ± 594	675.7 ± 258	1159.5 ± 584	1324.9 ± 609^‡^	0.001	1386.2 ± 670	1036.8 ± 538	1077.2 ± 439	1689.6 ± 712	< 0.001
CRP (0–0.5), (mg/dL)	5.1 ± 6.0	13.4 ± 6.3	4.8 ± 4.8	2.5 ± 4.6^‡^	< 0.001	3.1 ± 4.8	6.6 ± 6.4	3.9 ± 4.7	2.1 ± 4.4	0.005

Results are expressed as the mean ± standard deviation or as n (%).

*p values were analyzed by one-way ANOVA.

^†^No difference with other two clusters (p > 0.05 by post-hoc multiple comparison).

^‡^No difference with Cluster 2 (p > 0.05 by post-hoc multiple comparison).

*ALC* absolute lymphocyte count, *ANC* absolute neutrophil count, *CKD* chronic kidney disease, *CRP* C-reactive protein, *WBC* white blood cell.

SF ratio, SpO_2_/FiO2 ratio.

### Clinical outcome and oxygen requirement

**Figure 3:**
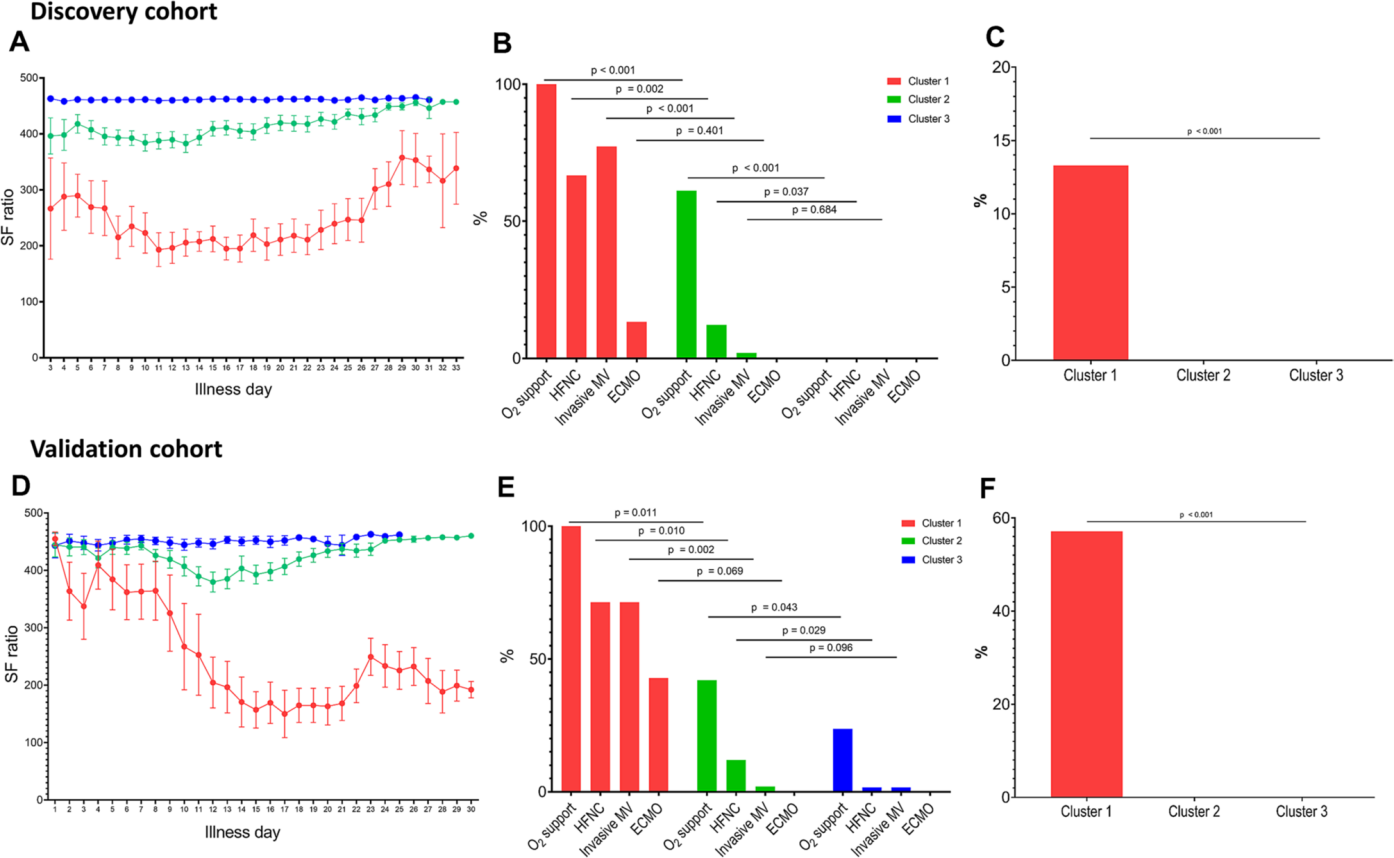
Association between three distinct patterns of inflammatory responses and disease severity in discovery cohort and validation cohort. Oxygenation status of the discovery cohort (**A**) and the validation cohort (**D**), expressed as the pulse oximetry saturation/fraction of inspired oxygen ratio (SF ratio) during hospitalization, depicted according to cluster. “Illness day” denotes the number of days from first symptom onset. Oxygenation support according to clusters in discovery cohort (**B**) and the validation cohort (**E**), Mortality according to clusters in discovery cohort (**C**) and the validation cohort (**F**). *ECMO* extracorporeal membrane oxygenation, *HFNC* high-flow nasal cannula, *MV* mechanical ventilator, *SF* ratio, pulse oximetry saturation/fraction of inspired oxygen ratio.

Figure 3A shows oxygenation status, defined by the SF ratio, during hospitalization according to the clusters. In Cluster 1, the SF ratio deteriorated continuously during the second week and remained low until end of Week 3. By the end of Week 3, oxygenation began to improve. Patients in Cluster 2 had poor oxygenation status during the first 2 weeks, but this recovered to normal by the end of Week 3. While no patients in cluster 3 required oxygen support, 100% of patients in Cluster 1 and 61% of patients in cluster 2 required oxygen support at some point during hospitalization (p-value < 0.001 on multiple comparison, Fig. 3B). Among patients who required supplement oxygen in Cluster 1, 11 (77.3%) required invasive mechanical ventilation and two (13.3%) required extracorporeal membrane oxygenation (ECMO). In Cluster 2, 6 (12.2%) patients required high flow nasal cannula and one (2.0%) patient required invasive mechanical ventilation. Two (13.3%) patients in Cluster 1 died; no patient in Clusters 2 and 3 died (Fig. 3C).

### Subgroup analysis according to age and risk factors

Older age is the single most important risk factor for an adverse outcome for COVID-19; therefore, we investigated immune responses in older (age ≥ 65 years) and younger (age < 65 years) patients. The clustering differed significantly between these age groups. Among 37 patients in the elderly group, 12 (32.4%) were grouped into Cluster 1 and only three (8.1%) were grouped into Cluster 3. By contrast, only three (4.4%) of the younger patients were grouped in Cluster 1, whereas 33 (48%) were included in Cluster 3. The percentage in Cluster 2 was similar for both age groups (Supplementary table S1). The SF ratio, CRP levels, and the ANC and ALC fell into three clusters for patients ≥ 65 years-of-age and < 65 years-of-age; the pattern was similar to that observed for the whole population, although CRP levels were generally higher and the ALC lower in the older group (Supplementary figure S1 and S2).

### Validation of the cluster analysis

The validation cohort included 116 patients with a mean age of 50.2 ± 18.6 years and 49 (59.0%) were men. The baseline characteristics and laboratory parameters on admission are summarized in Table 1. The patterns of changes in inflammatory markers (CRP, ANC and ALC) in 3 clusters were markedly similar to those from the discovery cohort, although the CRP levels and neutrophil counts in Cluster 1 of the validation cohort tended to be more close to the other clusters as compared to those in the discovery cohort (Fig. 2E–H). Strikingly, a similar association between clinical outcomes and the clusters was observed (Fig. 3D–F).

## Discussion

To the best of our knowledge, this study based on unbiased clustering analysis of CRP levels, neutrophil counts, and lymphocyte counts is the first to identify three different patterns of inflammatory immune response during COVID-19 infection. All patients in Cluster 1 (with high CRP levels, a high ANC, and a low ALC) had a severe clinical course and required oxygen support, whereas all patients in Cluster 3 (minimal CRP elevation, normal ANC, and normal ALC) showed the best clinical course, with no requirement for supplementary O_2_ and no deaths. Patients in Cluster 2 took an intermediate path with respect to inflammatory marker values and clinical course. The close association between the patterns of inflammatory immune response and clinical outcome was validated in a second independent cohort.

In a recent study a similar observation was made in sepsis patients. Using routine laboratory and clinical parameters the study identified 4 different sepsis phenotypes that were closely associated with host-response patterns and clinical outcomes^9^. The δ sepsis phenotype with the highest mortality was associated with elevated serum lactate levels and IL-6 compared with other sepsis phenotypes, supporting that host inflammatory response is associated with clinical outcome.

The immune response to infection is a dynamic process involving activation of humoral and cellular components, resulting in fluctuations in inflammatory marker expression over time^10,11^. Here, we analyzed longitudinal patterns (i.e., time series data) in the levels of common inflammatory markers (CRP levels, the ANC, and the ALC) over time rather than at a single time point in each individual. As expected, inflammatory markers showed dynamic changes over time, rising during the early phase of infection and falling during the recovery phase (Fig. 2). This supervised clustering analyses identified three distinct clusters (i.e., patterns) of immune response. Cluster 1 (hyperinflammatory immune response) was characterized by the high CRP levels, neutrophilia, and lymphopenia. Cluster 3 (hypo-inflammatory immune response) showed only mild to moderate increases in CRP, but normal neutrophil and lymphocyte counts. The inflammatory immune response in Cluster 3 is commonly observed during a “normal” viral infection, which is characterized by a relatively mild increase in CRP levels and mild leukopenia^12^ and none of the patients in Cluster 3 required oxygen and none died. By contrast, the additional increase in CRP and the accompanying neutrophilia were characteristic of the hyperinflammatory (overshooting) response in Cluster 1. This hyperinflammatory immune response mimics a macrophage activation syndrome (MAS) which is characterized by high CRP, neutrophilia, and hyperferritinemia^13–15^. MAS and severe COVID-19 infection share elevated CRP and hyperferritinemia along with increased interleukin (IL)-1β, IL-2, IL-6 and tumor necrosis factor^13^. Laboratory changes observed in Cluster 3 resemble the immune response of systemic lupus erythematosus (SLE), where type 1 interferon during active SLE mediates leukopenia, ESR elevation, and a modest increase in CRP^6,16^. However, a small subset of SLE patients develop a secondary and potentially lethal MAS characterized by a rapid increase in CRP, WBC, ferritin, and d-dimer levels^17^. These laboratory findings of MAS are similar to those observed for Cluster 1.

All patients in the hypoinflammatory Cluster 3 had a mild course without requiring O_2_, whereas all patients in Cluster 1 had severe COVID-19 disease and required O_2_ supplementation: 73.3% of patients required mechanical ventilation and 13.3% received ECMO. Of the patients in Cluster 2, 30 (61.2%) required oxygen supplementation, but only one (2.0%) required mechanical ventilation (Fig. 3). These findings were validated in a second independent cohort. This clearly shows that inflammatory immune responses are strongly associated with the severity and outcome of COVID-19. Therefore, it is not the SARS-CoV-2 virus, but rather the harmful hyperinflammatory host immune response to the virus that ultimately determines the outcome of those with COVID-19.

This striking difference in immune responses to the same virus highlights the importance of host factors. Consistent with prior findings, patients in Cluster 1 were older and were more likely to have hypertension^18^ (Table 1). Indeed, 12 (32.4%) and 17 (45.9%) of the 37 patients aged > 65 years-of-age were in Cluster 1 and Cluster 2, respectively. Only eight (21.6%) patients were in Cluster 3 (Supplementary table S2). This hyperinflammatory immune response in the elderly appears to contradict the wide assumption of age-associated immunosenescence in the elderly^19,20^. Immune cells from elderly patients with gout respond to urate-crystals in vitro by producing more inflammatory cytokines than those from younger patients with gout^21^. Of note, a stronger inflammatory response does not necessarily correlate with an effective immune response; for example, high CRP levels are inversely correlated with responses to vaccines^22,23^.

One strength of this study is the use of unbiased clustering analysis of the immune response. This approach eliminates the risk of subjective interpretation of patterns. Clustering was based only on the longitudinal pattern of inflammatory markers (without a priori clinical information such as age, hypertension, or treatment outcome), and the patterns were correlated with clinical course and outcome later. The analysis of longitudinal patterns of inflammatory markers enabled to group patients with similar laboratory values at admission into Cluster 2 and Cluster 3 with different clinical course (Table 1). Strikingly, the patients in Cluster 1 of the validation cohort had relatively low CRP levels and neutrophil counts at baseline. However, as the CRP and neutrophil count rose, the oxygen requirement increased, emphasizing not a single value (at admission) but the overall pattern of inflammatory response is more predictive of clinical outcome. This dynamic nature of immune response should be considered when developing a prediction model of an infectious disease including COVID-19.

This clustering analysis did not include the anti-microbial and immunomodulatory treatment or potential bacterial superinfection. The anti-viral agents were used in 20 (90.1%) out of 22 patients in Cluster 1, 61 (61.6%) of 99 patients in Custer 2 and 44 (44%) in 100 patients in Cluster 3 (Supplementary Figure S3A). Corticosteroids were used in 12 (54.5%) of 22 in Cluster 1, 3 (3.0%) of 99 in Cluster 2 and 2 (2%) of 100 patients in Cluster 3 (Supplementary Figure S3B). None of the patients received IL-6 or IL-1 blocking agents. In the patients with severe disease, empirical antibiotics were administered for a potential bacterial superinfection. However, in 4 patients in Cluster 1, the extensive microbiological studies including bronchoscopic washing and repeated blood cultures were negative, suggesting that the hyper-inflammatory response in cluster 1 cannot be fully explained by a possible bacterial co-infection alone. Procalcitonin (PCT), which is helpful distinguishing bacterial infection from non-bacterial infection^24^, was not included in the pattern analysis since they were measured only in a small number of patients. Although the overall inflammatory response to COVID-19 infection, possibly influenced by treatment and complications including bacterial superinfection, are roughly captured in common non-specific inflammatory markers, they are not able to fully reflect the complexity of inflammatory response in COVID-19 patients. More research is needed to determine whether multi-dimensional approaches with a larger number of inflammatory markers and cytokines might better cluster the inflammatory responses in COVID-19 patients. Lastly, further studies are needed to assess whether serial changes in initial laboratory markers predict long-term outcomes.

In conclusion, unbiased clustering analyses identified distinct patterns of immune response to SARS-CoV-2 infection. Hyperinflammatory immune responses with elevated CRP levels, neutrophilia, and lymphopenia are associated with severe disease and a worse outcome. Further study is needed to investigate whether targeting the overshooting inflammatory responses can improve the clinical outcome of those with COVID-19.

## Methods

### Patients and data collection

From 24 January 2020 to 14 April 2020, 130 consecutive patients who were diagnosed with COVID-19 and admitted to one of three medical centers (National Medical Center (NMC), Seoul National University Bundang Hospital (SNUBH), and Seoul National University Hospital (SNUH)) were recruited retrospectively in a discovery cohort. From 2 February 2020 to 10 July 2020, 116 of 150 consecutive patients from 4 hospitals in Korea who were treated at NMC (n = 50), Armed Forces Capital Hospital (n = 41), Myongji Hospital (n = 40) and (4) SNUH (n = 19) were included into a second independent validation cohort (Fig. 1). These medical centers are the designated hospitals for COVID-19 treatment in Korea.

A diagnosis of COVID-19 was based on detection of SARS-CoV-2 in respiratory samples by real-time reverse transcriptase–polymerase chain reaction performed at the participating institutions or at the Korea Centers for Disease Control and Prevention. Information about baseline demographics, clinical characteristics, vital signs, and laboratory test results were obtained from electronic medical records. The blood samples were collected on admission day and during hospitalization at the discretion of the treating physicians. No additional blood samples were obtained for research purposes.

The study was conducted in accordance with the principles of the Declaration of Helsinki and Good Clinical Practice guidelines. The study was approved by the Institutional Review Board of each participating center (NMC, SNUBH, SNUH, Armed Forces Capital Hospital and Myongji Hospital). The Institutional Review Board of each participating center (NMC, SNUBH, SNUH, Armed Forces Capital Hospital and Myongji Hospital) waived the informed consent as the study involved minimum risk to the patients and no identifiable information was used.

### Oxygenation status

Oxygen saturation (as measured by pulse oximetry saturation (SpO_2_) and fraction of inspired oxygen (FiO_2_)) were retrieved from electronic vital sign sheets. The lowest SpO_2_ value and the highest FiO_2_ value on a given day (from 00:00 to 24:00) were used to calculate the daily SpO_2_/FiO2 ratio (SF ratio). The FiO_2_ on room air was set as 0.21.

### Imputation of missing values

Missing values were imputed using linear interpolation between the non-missing values immediately before and after the missing time point, with a calculated variation designed to follow the shape of the average trajectory in a population^25^.

### Supervised clustering: k-means clustering

Time-series of CRP, ANC, and ALC measures were used for supervised k-means clustering. Although IL-6, ferritin, lactate dehydrogenase and d-dimer among others have also been reported to be associated with clinical course of COVID-19, these markers are not commonly available in patient with COVID-19 infection^18,26^. Since patients can have a mild, moderate, or severe (life-threatening) course, we performed supervised clustering analysis to group COVID-19 patients into three clusters based on longitudinal measurement of CRP, ANC, and ALC.

Dynamic time warping (DTW), a dynamic programming algorithm that compares two series and finds the optimum warping path between them, was utilized for cluster analysis. DTW enables a distance calculation between two time series of different lengths while minimizing the effects of shifting and distortion in time by allowing elastic transformation of a time series to detect similar shapes^27^. For k-means clustering, objects were grouped by gathering around the mean/centroids of each k cluster. A DTW distance based centroids identification algorithm, DTW barycenter averaging, was used^28^. For each time point in the centroid, all corresponding values from all series in the cluster were grouped together according to the DTW alignments and the mean was calculated for each centroid point using the values contained in each cluster. This centroid update process was repeated until convergence was reached and a k number of clusters was identified. The cluster analysis was performed independently in the discovery cohort and validation cohort.

### Statistical analysis

Normally distributed continuous variables were expressed as the mean ± standard deviation, and groups were compared using one-way analysis of variance (ANOVA) and post hoc multiple comparison using Tukey and Dunnett’s test. The Chi-square or Fisher’s exact test was used to compare categorical variables. All analyses were performed using Rstudio (version 1.2, Boston MA, USA) and SPSS (IBM SPSS Statistics for Windows, Version 25.0). Clustering of immune responses was processed using R package ‘longitudinalData’ and ‘dtwclust’. A *P*-value < 0.05 was considered statistically significant.

## Supplementary Information

Supplementary Information.
