# Supplementary material for: Pattern of inflammatory immune response determines the clinical course and outcome of COVID-19: unbiased clustering analysis
Source: Sci Rep. 2021 Apr 13;11:8080. doi: 10.1038/s41598-021-87668-z (PMC8044143; doi:10.1038/s41598-021-87668-z)
Supplement: Supplementary file 1 — Supplementary Information. [file 41598_2021_87668_MOESM1_ESM.docx]

*Original article*

**Pattern of inflammatory immune response determines the clinical course and outcome of COVID-19: Unbiased clustering analysis.**

Eunyoung Emily Lee, MD^a*^, Kyoung-Ho Song, MD, PhD^b*^, Woochang Hwang, PhD^c^, Sin Young Ham, MD^b^, Hyeonju Jeong, MD^b^, Jeong-Han Kim, MD^d^, Hong Sang Oh, MD, MPH^d^, Yu Min Kang, MD^e,f^, Eun Bong Lee, MD, PhD^g^, Nam Joong Kim, MD, PhD^h^, Bum Sik Chin, MD, PhD^i**^, and Jin Kyun Park, MD, PhD^g**^

^a^Division of Rheumatology, Department of Internal Medicine, Uijeongbu Eulji Medical Center, Gyeonggi-do, Korea, ^b^Division of Infectious Diseases, Department of Internal Medicine, Seoul National University Bundang Hospital, Gyeonggi-do, Korea, ^c^Data Science for Knowledge Creation Research Center, Seoul National University, Seoul, Korea, ^d^Division of Infectious Diseases, Department of Internal Medicine, Armed Forces Capital Hospital, Gyeonggi-do, Korea, ^e^Department of Infectious Diseases, Myongji hospital, Gyeonggi-do, Korea, ^f^Department of Medical Education, Seoul National University College of Medicine, ^g^Division of Rheumatology, Department of Internal Medicine, Seoul National University Hospital, Seoul, Korea, ^h^Division of Infectious Diseases, Department of Internal Medicine, Seoul National University Hospital, Seoul, Korea, ^i^Division of Infectious Diseases, Department of Internal Medicine, National Medical Center, Seoul, Korea

*Both authors contributed equally to the work.

****Corresponding authors:**

**Jin Kyun Park, MD, PhD**

Division of Rheumatology, Department of Internal Medicine, Seoul National University Hospital, 101 Daehak-ro, Jongno-gu, Seoul, 03080, South Korea

E-mail: jinkyunpark@gmail.com; Telephone: +82-2-2072-4765; Fax: +82-10-2072-3198

**Bum Sik Chin, MD, PhD**

Division of Infectious Diseases, Department of Internal Medicine, National Medical Center, Euljiro 245, Jung-gu, Seoul, 04564, Seoul, Korea.

E-mail: moberrer@nmc.or.kr; Telephone: +82-22262-4748; Fax: +82-2-2268-0803

**Supplementary Figure S1.** Unbiased clustering of immune responses in patients ≥ 65 years old.


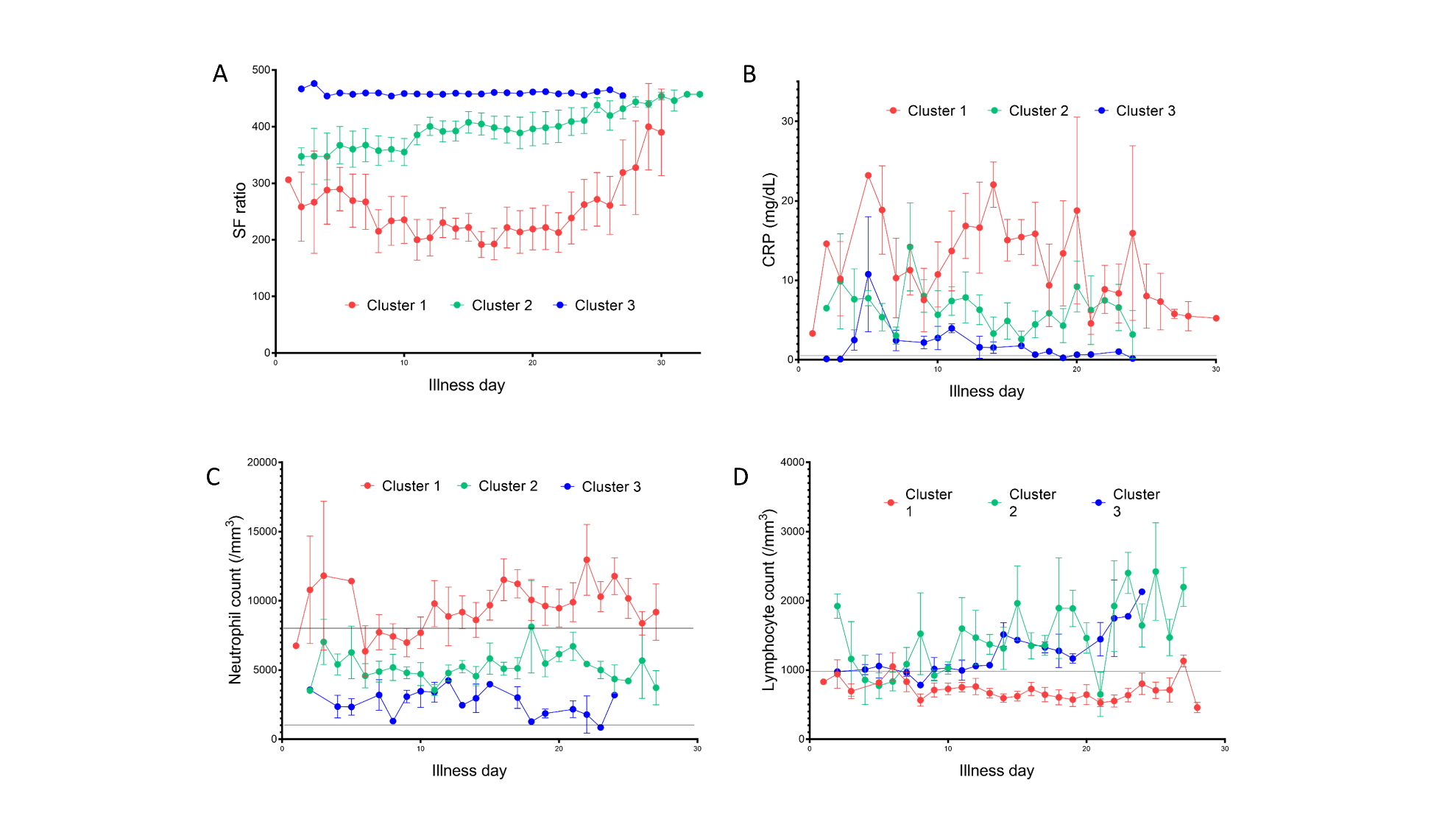


(A) Changes of SF ratio (B) Changes of C-reactive protein (C) Changes of absolute neutrophil counts (C) Changes of absolute lymphocyte count in each cluster. Illness day indicates days from the first symptom onset. Horizontal bars indicate normal range of the values. CRP, C-reactive protein; SF ratio, pulse oximetric saturation/fraction of inspired oxygen ratio.

**Supplementary Figure S2.** Unbiased clustering of immune responses in patients < 65 years old.

**
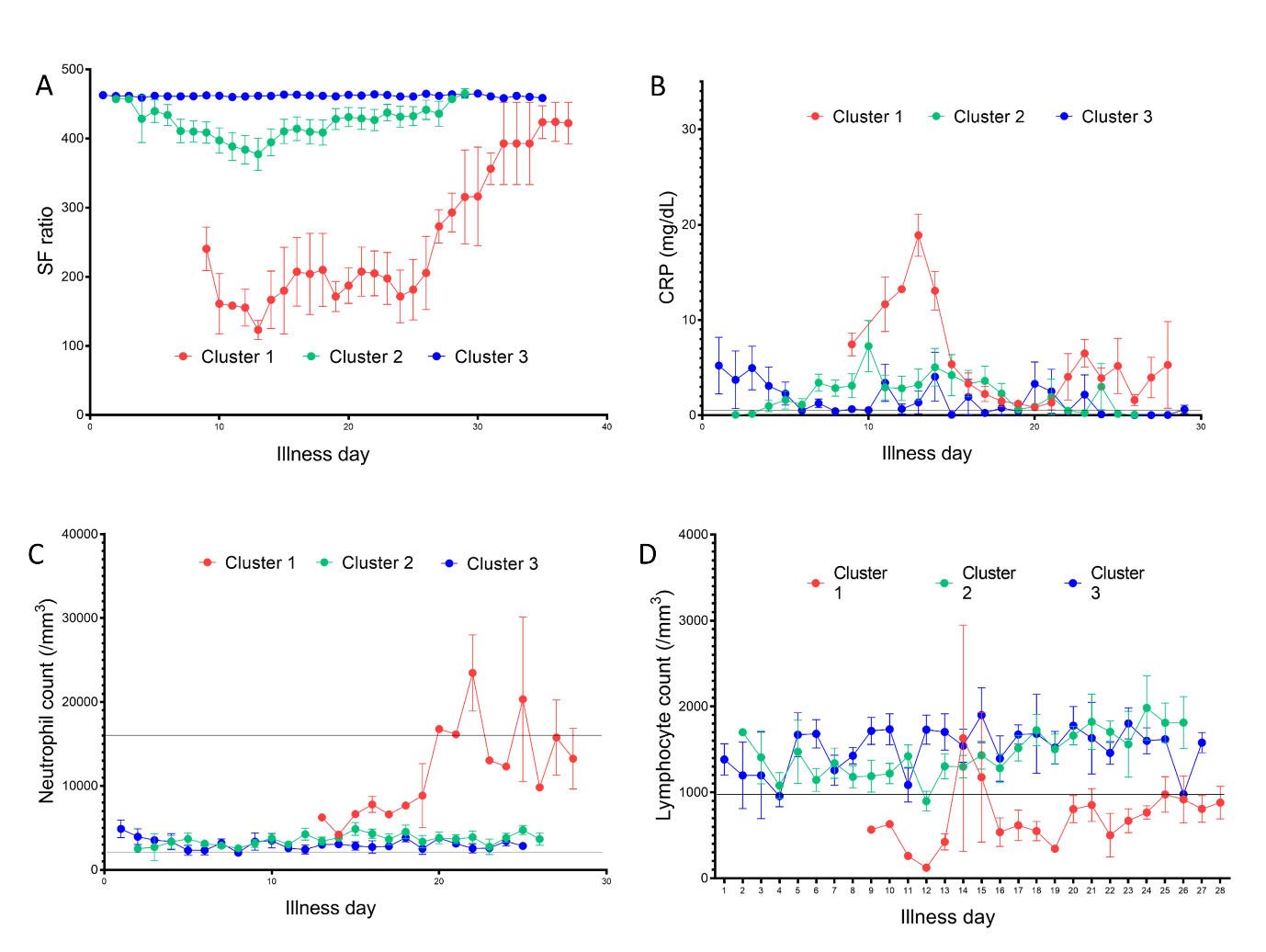
**

(A) Changes of SF ratio (B) Changes of C-reactive protein (C) Changes of absolute neutrophil counts (C) Changes of absolute lymphocyte count in each cluster. Illness day indicates days from the first symptom onset. Horizontal bars indicate normal range of the values. CRP, C-reactive protein; SF ratio, pulse oximetric saturation/fraction of inspired oxygen ratio.

**Supplementary figure S3**. Use of anti-viral agents (A) and corticosteroids (B) in each group.

**A.** **Antiviral agents**


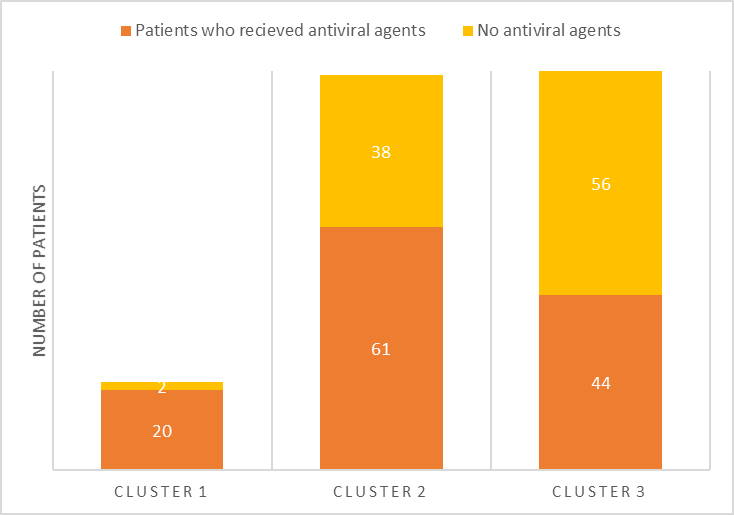


**B. Corticosteroids**

**Supplementary Table S1. Clinical outcomes (by age group)**

|  | Discovery cohort | | | Validation cohort | | |  |
| --- | --- | --- | --- | --- | --- | --- | --- |
|  | Age <65 years  (N=68) | Age ≥65 years  (N=37) | *P*-value | Age <65 years  (N=83) | Age ≥65 years  (N=33) | *P*-value | |
| Cluster, n (%) |  |  | <0.001^*^ |  |  | 0.003^*^ | |
| Cluster 1 (hyper-inflammatory) | 3 (4.4) | 12 (32.4) |  | 2 (2.4) | 5 (15.2) |  | |
| Cluster 2 (intermediate) | 32 (47.1) | 17 (45.9) |  | 32 (38.6) | 18 (54.5) |  | |
| Cluster 3 (hypo-inflammatory) | 33 (48.5) | 8 (21.6) |  | 49 (59) | 10 (30.3) |  | |
| Supplemental oxygen, n (%) | 19 (27.9) | 26 (70.3) | <0.001^*^ | 18 (21.7) | 24 (72.7) | <0.001^*^ | |
| High flow nasal cannula, n (%) | 6 (8.8) | 10 (27.0) | 0.013^*^ | 5 (6.0) | 7 (21.2) | 0.036^**^ | |
| Mechanical ventilator, n (%) | 3 (4.4) | 9 (24.3) | 0.002^*^ | 3 (3.6) | 4 (12.1) | 0.100^**^ | |
| Extracorporeal membrane oxygenation, n (%) | 1 (1.5) | 1 (2.7) | 1.000^**^ | 1 (1.2) | 2 (6.1) | 0.194^**^ | |
| Death, n (%) | 0 | 2 (5.4) | 0.122^**^ | 1 (1.2) | 3 (9.1) | 0.069^**^ | |

Results are presented as n (%). P values were generated by the ^*^Chi-square test or ^**^Fisher’s exact test.

**Supplementary Table S2. Clinical outcomes of the patients in the discovery cohort and the validation cohort (according to the clusters)**

**A. Discovery cohort**

|  | Total | Cluster 1(n=15) | Cluster 2 (n=49) | Cluster 3 (n=41) | *p*-value |
| --- | --- | --- | --- | --- | --- |
| Length of hospital day | 19.0 [15.0;20.0] | 20.0 [20.0;27.5] | 17.0 [14.0;20.0] | 20.0 [18.0;20.0] | 0.001 |
| Oxygen support, n (%) | 45 (42.9) | 15 (100.0) | 30 (61.2) | 0 | <0.001 |
| HFNC | 16 (15.2) | 10 (66.7) | 6 (12.2) | 0 | < 0.001 |
| Invasive MV | 13 (12.4) | 11 (73.3) | 1 (2.0) | 0 (0.0) | <0.001 |
| ECMO | 2 (1.9) | 2 (13.3) | 0 (0.0) | 0 (0.0) | <0.001 |
| Mortality | 2 (1.9) | 2 (13.3) | 0 | 0 | < 0.001 |

|  | Total | Cluster 1 (n=7) | Cluster 2 (n=50) | Cluster 3 (n=59) | *p*-value |
| --- | --- | --- | --- | --- | --- |
| Length of hospital day | 15.0 [14.0;23.5] | 28.0 [25.5;31.5] | 17.5 [14.0;22.0] | 14.0 [14.0;22.0] | 0.001 |
| Oxygen support, n (%) | 42 (36.2) | 7 (100.0) | 21 (42.0)* | 14 (23.7)* | < 0.001 |
| HFNC | 12 (10.3) | 5 (71.4) | 6 (12.0)* | 1 (1.7)* | < 0.001 |
| Invasive MV | 7 (6.0) | 5 (71.4) | 1 (2.0)* | 1 (1.7)* | < 0.001 |
| ECMO | 3 (2.6) | 3 (42.9) | 0* | 0* | < 0.001 |
| Mortality | 4 (3.4) | 4 (57.1) | 0 | 0 | < 0.001 |

**B. Validation cohort**

*p>=0.05. ECMO, extracorporeal membrane oxygenation; HFNC, high flow nasal cannula; MV, mechanical ventilation.
